# Increasing equitable access to telehealth oncology care in the COVID‐19 National Emergency: Creation of a telehealth task force

**DOI:** 10.1002/cam4.5176

**Published:** 2022-10-10

**Authors:** Brooke Worster, Lauren Waldman, Gregory Garber, Tingting Zhan, AnaMaria Lopez, Olivia Trachtenberg, Nathan Handley, Kristin L. Rising, Valerie Csik, Amy Leader

**Affiliations:** ^1^ Sidney Kimmel Cancer Center Thomas Jefferson University Hospital Philadelphia Pennsylvania USA; ^2^ Jefferson Health New Jersey Sewell New Jersey USA; ^3^ Department of Medical Oncology Thomas Jefferson University Philadelphia Pennsylvania USA; ^4^ Center for Connected Care Thomas Jefferson University Philadelphia Pennsylvania USA; ^5^ Division of Biostatistics, Department of Pharmacology & Experimental Therapeutics Thomas Jefferson University Philadelphia Pennsylvania USA; ^6^ Department of Emergency Medicine Sidney Kimmel Medical College, Thomas Jefferson University Philadelphia Pennsylvania USA; ^7^ College of Nursing, Thomas Jefferson University Philadelphia Pennsylvania USA

**Keywords:** clinical oncology, COVID‐19 pandemic, healthcare disparities, health literacy, qualitative research, telemedicine

## Abstract

**Introduction:**

Telehealth (TH) utilization in cancer care prior to COVID‐19 was variable. Research highlights disparities in access determined by socioeconomic factors including education, income, race, and age. In response to COVID‐19 and these disparities, we assessed the impact of a personalized digital support structure, the Telehealth Task Force (TTF), to reduce disparities in TH.

**Methods:**

We performed a retrospective review of cohorts between January 1, 2020 and August 30, 2020: Pre (TH use with basic telephone support), Intervention (TH access with TTF), and Post (TH access after TTF initiation and educational material dissemination). Data collected included successful TH access, health literacy (HL), and Area Deprivation Index, a ranking of neighborhoods by socioeconomic disadvantage (ADI). The data were analyzed in univariate ordinary least squares model and adjacent categories ratio model using statistical software R to understand the relationship between TTF, HL, ADI, and TH access.

**Results:**

We included 555 patients from January 1, 2020 to August 30, 2020 (90 preintervention, 194 intervention, and 271 postintervention), excluding patients without ADI/HL. TTF support successfully engaged older, racially, and socioeconomically diverse patients in TH; ADI is significantly higher in the postintervention group vs. preintervention (mean difference = 7.66, 95% CI 1.00–4.32, *p* = 0.024) and more patients had low HL during intervention compared with preintervention (adjacent categories ratio = 0.62, 95% CI 0.41–0.93, *p* = 0.021).

**Discussion:**

COVID‐19 created an immediate need for TH. Implementation of the TTF helped close the digital divide, increasing TH access for vulnerable patients. Attention to digital readiness can mitigate disparities in access to care. Future research should explore the implementation of widespread routine digital support initiatives.

## Graphical abstract

In response to COVID‐19 and disparities in access determined by socioeconomic factors, we assessed the impact of a personalized digital support structure, the Telehealth Task Force (TTF), to reduce disparities in TH. Implementation of the TTF helped close the digital divide; increasing TH access for vulnerable patients. Attention to digital readiness can mitigate disparities in access to care.

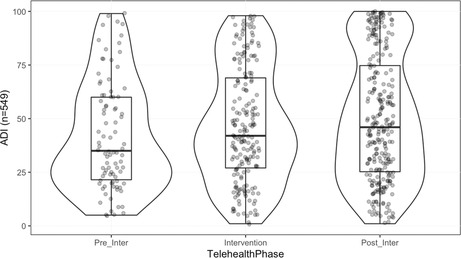

## 1 INTRODUCTION

There is a general increase in drive among oncology practices to incorporate modern communication technology into the cancer care continuum. A hallmark of the COVID‐19 pandemic has been the rapid uptake of telehealth (TH), defined by the Centers for Medicare and Medicaid Services as the ability to talk with a clinician live via phone or video chat, send, and receive e‐messages with a clinician, or use remote monitoring so a clinician can assess a patient at home.^1^ Prior to the COVID‐19 pandemic, it was uncommon for United States healthcare systems or individual providers to utilize telehealth (TH) for routine clinical care, though rates were steadily increasing.^2^ A 2020 study on the rise of virtual care reported TH use was generally low and even in health systems with relatively high adoption of TH in 2019 and early 2020 prior to the “Stay‐at‐Home” orders implemented in response to COVID, had around 100 visits per day, paling in comparison to pandemic TH visit rates of 600 to 1000 per day.^3^ One institution found that their TH use increased from less than 1% of total visits to 70% of total visits at the beginning of the COVID‐19 pandemic.^4^ In part, this meteoric increase resulted from local and national limitations on state‐to‐state travel and in person encounters, and relaxation of HIPPA and billing regulations.^5^

Digital technology has shown great promise in improving healthcare, but there are system‐wide and individual‐level disparities that may deter patients from receiving this type of care.^6^ The surge in TH use as a result of the COVID‐19 pandemic illuminated vulnerabilities in access to telehealth that mirror many of the socioeconomic factors that affect health outcomes in general, as those with older age, low socioeconomic status, racial and ethnic minorities, lack of broadband access, and low health literacy, defined by the National Institute of health as the degree to which individuals have the ability to find, understand, and use information and services to inform health‐related decisions and actions for themselves and others and less social support struggled to adopt TH.^7^, ^8^ Prior to COVID‐19, patients with cancer who were more engaged in TH and digital health interventions were largely younger and more affluent.^9^ Disparities in both access and digital health literacy, an extension of health literacy but in the context of technology, disproportionally affect vulnerable populations, which will not improve without identifying and addressing access and uptake challenges.^10^, ^11^ Prior research conducted by our team revealed that our patients with a high school diploma or less or who identified as a racial or ethnic minority were less likely to access the internet, indicating a need for targeted interventions to address these disparities.^12^

Although the disparity in accessing TH is a known concern, there is less information about the digital access and preferences of patients with cancer as a population. This is an especially important population to be able to both avoid treatment delays and shield themselves from infectious risks. Patients who perceive their care to rely heavily on physical examination and diagnostic testing may have concerns about receiving the standard of care to which they are accustomed via TH.^13^ Patients with disparate traits including lower health literacy, driven by older age, lower socioeconomic status, minority race or ethnicity, mistrust of technology, lower level of education may have increased distress related to TH.^14^, ^15^, ^16^ The purpose of this retrospective cohort study is to assess the impact of a personalized, real‐time support structure, the Telehealth Task Force (TTF), which was implemented during the COVID‐19 pandemic to improve the uptake of TH, defined here as a live audio‐visual clinician appointment, among a diverse population of vulnerable cancer patients. With this work, we aim to inform the development of future initiatives focused on reducing patient‐level barriers to digital access to cancer care. Specifically, we look to address the structure needed to increase successful access to TH in patients with low health literacy and/or socioeconomic disadvantage.

## 2 METHODS

### 2.1 Study design

We conducted a retrospective cohort study from January 1, 2020 to August 30, 2020 to assess the impact of a telehealth task force created in a cancer center (TTF) on patient use of TH. The TTF was created during the COVID‐19 pandemic and was continued through December 10, 2020. This study compares outcomes across patients with cancer seeking care pre, during, and post‐TTF intervention periods.

### 2.2 Setting

Jefferson Health is an 18‐hospital academic health system spanning two states and is the largest health system in the Philadelphia region. Jefferson Health began using TH in 2017 to provide both urgent care and ongoing primary and specialty care access to a tertiary medical center.^17^ The NCI‐designated Sidney Kimmel Cancer Center (SKCC) is the cancer research and clinical care arm of Jefferson Health. The SKCC catchment area is highly diverse, with significant health and cancer disparities across the region.^18^ The SKCC sees more than 9000 new cancer diagnoses annually. The Neu Center for Supportive Medicine and Cancer Survivorship (NCSM) is The SKCC's outpatient interdisciplinary palliative care team. The NCSM sees any patients regardless of age, cancer type/stage, or treatment plan. Patients enter into the NCSM by referral or through a routine SKCC‐wide distress screening, which includes the HL questionnaire.

### 2.3 Sample

Research team members utilized electronic health records to identify patients being seen at the SKCC for treatment of their cancer and who had completed at least one TH visit from January 1, 2020 to August 30, 2020. Any patients with cancer seen in our cancer center during the time frame January 1, 2020 to August 30, 2020 who completed at least one TH visit were eligible to be included in this analysis. Patients with successful TH and completed health literacy information between January 1, 2020 and March 15, 2020 were considered pre‐TTF as these encounters took place prior to the peak of the COVID‐19 pandemic and the creation of the TTF. Patients seen between March 16, 2020 and June 7, 2020 were considered the TTF Operational period and those seen via TH between June 8, 2020 and August 30, 2020 were considered post‐TTF. The transition from fully operational TTF period to post‐TTF occurred because COVID‐19 case rates were falling, increased understanding of infection prevention was implemented in the health system and more in‐person visits were possible. These patients had varied diagnoses: solid tumor stages 1–4 or varied types of hematologic malignancies such as lymphomas, acute, and chronic leukemia as well as multiple myeloma were referred to and assisted by the TTF.

### 2.4 Intervention

**FIGURE 1:**
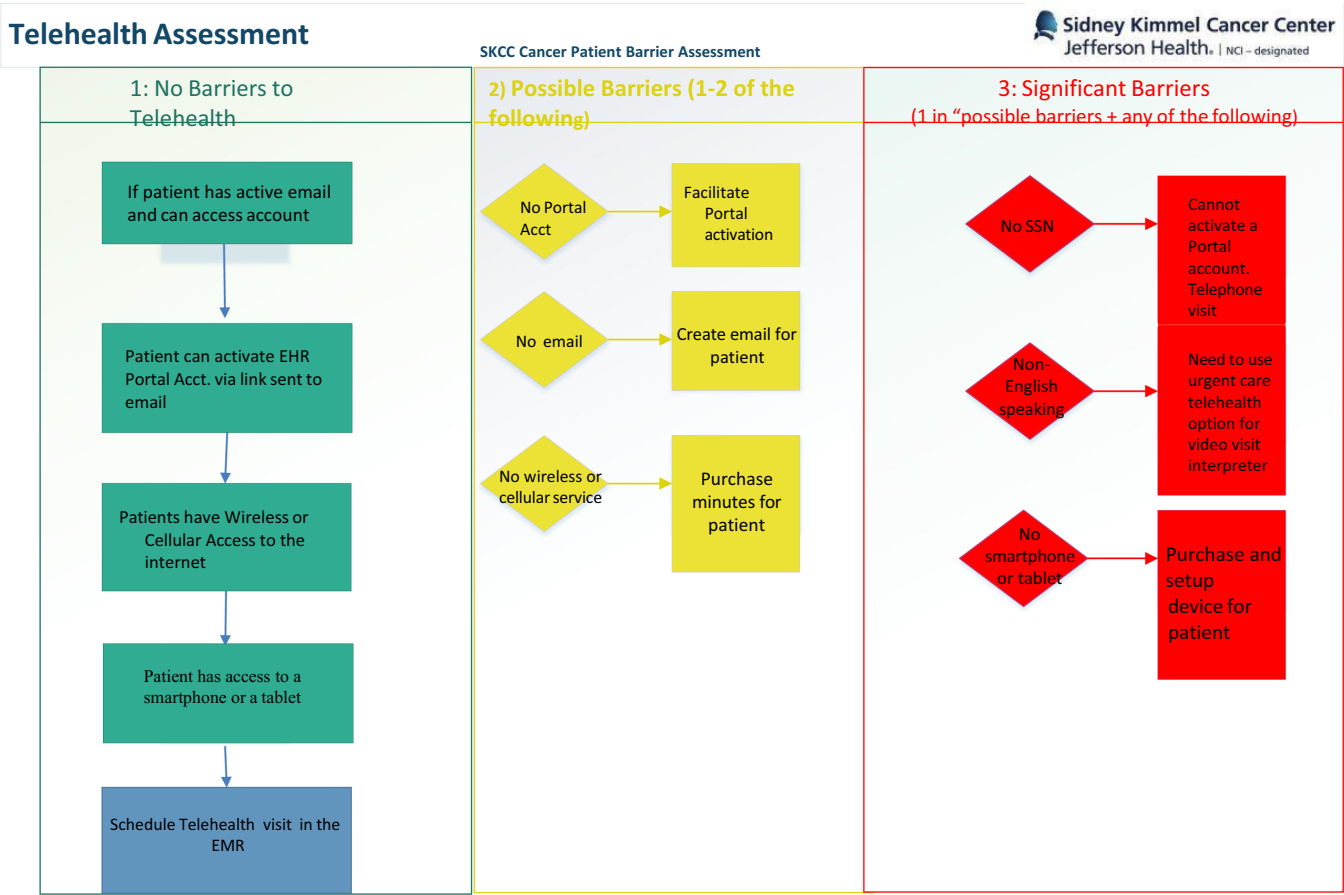
Telehealth assessment: Workflow for telehealth barrier assessment and possible solutions

Prior to COVID‐19, utilization of TH was available to all patients who were able to or were interested in using the service, with the institution providing basic telephone‐based support to patients in need of assistance. If the patient took it upon themselves to call a help line. Resulting of the COVID‐19 pandemic and an urgent need to keep our patients on treatment and reduce risk, the TTF was created and deployed across the SKCC in March 2020. The TTF consisted of individual outreach with a personalized intervention based on patient‐level needs **(**Figure 1 **)**.

It comprised a range of staff including graduate students, project managers, and research coordinators who had no prior expertise in technology education. The Interprofessional leadership team in charge of developing this resource consisted of a physician, a social worker, and a project manager. They consulted with the team leading TH efforts across the healthcare system prior to COVID‐19. This consisted of a nurse, IT, and care coordinators with extensive technology experience. The TTF intervention could include any of the following, depending on patient needs: provision of a smart‐device and service, access to broadband internet, audio or visual one‐on‐one education about how to download a smart device app, access to the Jefferson Health patient health portal, creation of email accounts, and” practice” visits to aid in the successful use of TH for cancer care and communication.

The interventions needed were developed iteratively with an initial list of ‘common problems’ drawn from the health system TH team and added to when new problems or barriers were uncovered in the course of patient care. Patients could have had any number of these services, depending on the barrier to TH they identified. Patients were identified for TTF assistance either by self‐referral, clinical referral, or missed appointments. When a patient missed a TH call, care coordinators reached out via phone and if the barrier was related to TH, the referral to TTF was made. The TTF served not only to engage patients but also to create educational materials to help staff and care teams educate patients with technology barriers.

### 2.5 Measures

Health literacy was assessed via the BRIEF questionnaire.^19^ The BRIEF health literacy questionnaire is a 4‐item survey with a 5‐point Likert scale including items such as, “How often do you have someone help you read hospital materials?” scored from always (1), often (2), sometimes (3), occasionally (4), never (5), and “How confident are you filling out medical forms by yourself?” with answers of not at all (1), a little bit (2), somewhat (3), quite a bit (4), or extremely (5). Scores of 4–12 indicate low health literacy, 13–16 indicate medium health literacy, and 17–20 indicate high health literacy. This tool takes fewer than 5 min to complete. The final score was entered into the patient's electronic health record (EHR). Missing health literacy information was obtained by outreach from research assistants, who attempted to contact patients twice, providing return call information if unable to reach patients.

Researcher assistants compiled patients' addresses from the EHR to collect Area Deprivation Index (ADI) scores.^20^ The ADI is a tool used to describe socioeconomic disadvantage and considers socioeconomic factors such as; income, education, employment, and housing quality. Results range from 0 to 100, with higher scores indicating higher levels of socioeconomic disadvantage. Previous studies have used the ADI to understand community needs and to prioritize interventions and general healthcare delivery.^21^, ^22^ The primary outcome was the completion of at least one successful audio‐visual telemedicine appointment. The BRIEF HL tool and ADI were used in this analysis as we collect the BRIEF HL on all patients as a standard of care and the ADI can be calculated using each patient's home address. Given that this was a real‐time sample of patients during the COVID‐19 pandemic, we could not introduce additional measures meant solely for research purposes within the entire population of our cancer center.

### 2.6 Statistical analysis

Descriptive and demographic statistics were summarized for each of the three study phases. The relationship between ADI and study phase (preintervention, intervention, and postintervention) is analyzed based on 549 patients by fitting a univariate ordinary least squares model using statistical software R.^23^ The relationship between HL and study phase is analyzed based on 469 patients by fitting a univariate adjacent‐categories‐ratio model using R package VGAM.^24^ Examining the health literacy and socioeconomic disadvantage of patients connecting to TH prior to the TTF and after the TTF available illuminates what is needed to support vulnerable patients with cancer.

This study was approved by the Thomas Jefferson University Institutional Review Board to be exempt from consenting patients, as the data collected were all a part of routine patient care.

## 3 RESULTS

### 3.1 Study population

A total of 555 patients were identified from medical records. Several patients were excluded from analysis due to having missing ADI, as their residence lays within an area that does not have an ADI (i.e., commercial/government area in Center City Philadelphia, PA). Patients who were unable to be reached by telephone to complete the health literacy questionnaire or who were deceased at the time of data retrieval were excluded from relevant analyses, with 86 participants being excluded in total (preintervention, *n* = 17; intervention, *n* = 21; and postintervention, *n* = 48). The total included in the analysis was 469. Patients were included regardless of geographic location.

Patients were slightly more female (53%) than male (47%), the average age of 63.9 years old (range: 28–89), and had a diverse racial representation: Black (35%), White (59%), and almost 6% other races. Roughly, 16.9% of the SKCC patient population is Black. Only 3% of patients identified as Hispanic or Latino vs 93% not Hispanic or Latino with 4% declining to answer.

### 3.2 Telehealth visits

During the preintervention period, 0.52% of the 15,126 scheduled appointments at the SKCC were TH (*n* = 79). During intervention, 25.57% of the 16,764 scheduled appointments were TH (*n* = 4287). During postintervention, 19.85% of the 17,334 scheduled appointments were TH (*n* = 3446). The dramatic increase from less than 1% of patients being seen via TH to close to one‐quarter of patients being seen in this fashion was driven by the “Stay‐at‐Home” orders and the pandemic, rather than an attempt to change clinical care without this force. Nonetheless, this is a profound shift in day‐to‐day care in a cancer center than happened within a very short time span. In total, 1127 patients were assisted by the TTF from March 16, 2020 to December 12, 2020, with 596 of these interventions occurring during the TTF Intervention period.

Of the 469 patients included in this analysis, 90 were preintervention, 194 were during the intervention period, and 271 were postintervention. One hundred percent of patients individually assisted by the TTF successfully completed at least one synchronous audio‐visual TH encounter with an oncology clinician.

### 3.3 Interaction between TTF, HL, and ADI

Successful engagement with TH in older adults significantly increased with the support of a TTF. The majority of patients across all time points were 55 or older (*n* = 440). Slightly more patients self‐identified as female (*n* = 294) than as male (*n* = 261). Only 27 (30%) patients in the preintervention cohort were 65 or older, whereas 137 (50%) in the postintervention cohort were 65 or older (*p* < 0.001).

**FIGURE 2:**
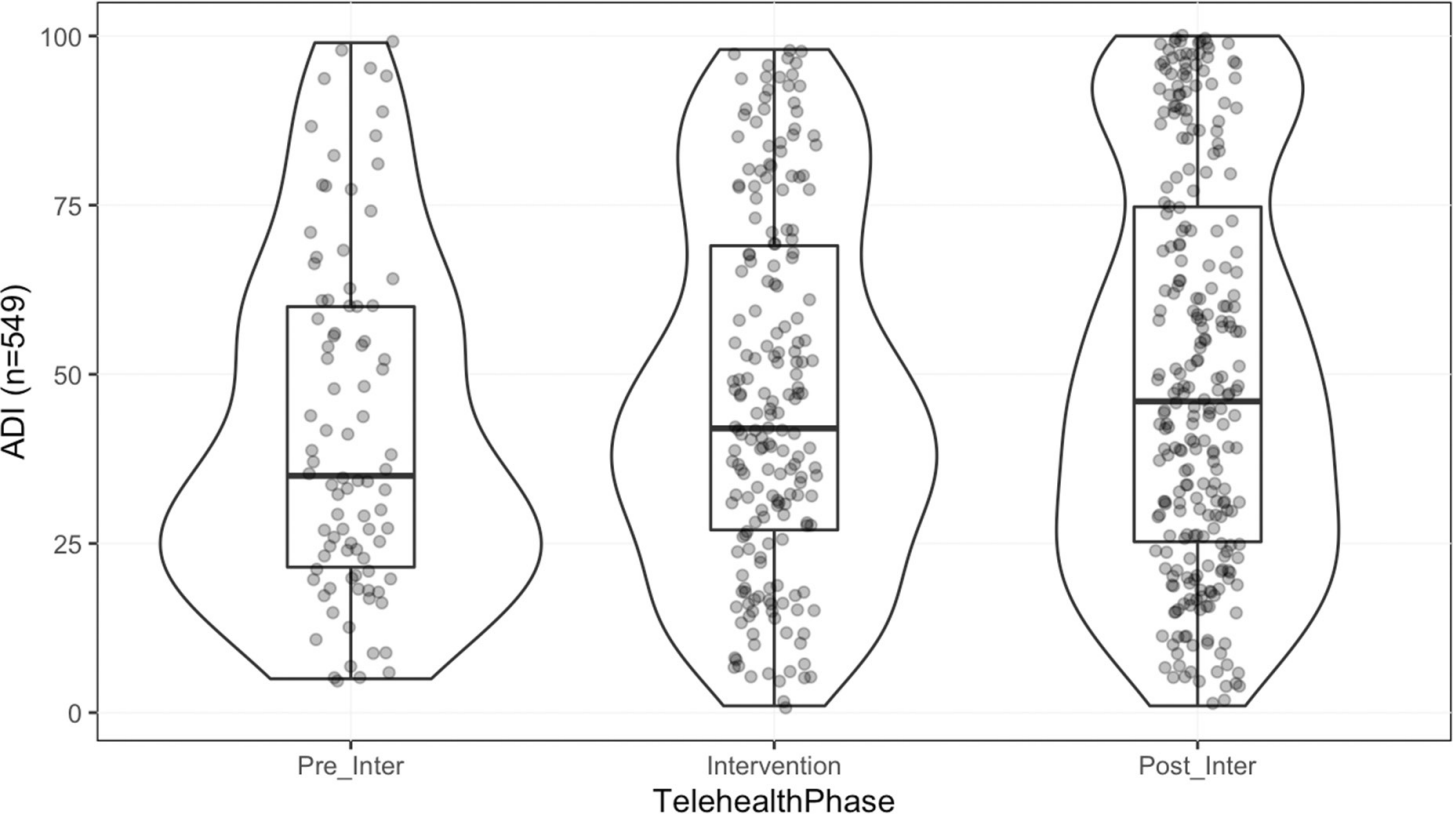
Area deprivation index (ADI) score by study phase. Scores range from 0 to 100 with higher scores representing more socioeconomic deprivation. Each depiction of the study phase includes a box plot where the outer boundaries of the box represent standard deviation, and the bolded center‐line represents the median. The curved lines around the box plot represent the distribution; a bulbous area therefore demonstrates a disparate distribution.

Fifty‐nine percent of patients identified as White/Caucasian and 35% identified as Black/African American (*n* = 325, *n* = 193), with few patients identifying as Asian (*n* = 10) and Hispanic (*n* = 18). Telehealth utilization significantly increased among Black patients with the TTF intervention (25% to 40%, *p* < 0.001) (Table 1). The TTF engaged patients with a higher ADI, indicative of more patients with a greater socioeconomic disadvantage being able to participate in TH with the support of the task force (mean difference = 7.66, 95% CI [1.00–14.32], *p* = 0.024) (Table 2 and Figure 2). In addition, the TTF increased successful access to TH in patients with low health literacy(adjacent categories ratio = 0.62, 95% CI 0.41–0.93, *p* = 0.021). Prior to the intervention, 6.7% of patients with low HL (6/90) successfully completed a TH visit, whereas 17.0% of low HL (33/194) completed one during the intervention period (Table 3).

**TABLE 1:** Cohort characteristics and effect of telehealth task force (TTF) on successful telehealth utilization

	*N* = 555	Preintervention *N* = 90 (16.2%)	Intervention *N* = 194 (35.0%)	Postintervention *N* = 271 (48.8%)	Significance level
Gender: *n* (%)					0.097 (Intervention vs. Pre_inter) *0.031 (Post_inter vs. Pre_inter) 0.663 (Post_inter vs. intervention) (Pairwise proportion test)
Female	294 (53.0%)	38 (42.2%)	104 (53.6%)	152 (56.1%)	
Male	261 (47.0%)	52 (57.8%)	90 (46.4%)	119 (43.9%)	
Age *n* (%)					
Age ≤ 35 years	17 (3.1%)	6 (6.7%)	5 (2.6%)	6 (2.2%)	*0.000 (Pearson's *χ* ^2^)
35 < Age ≤ 45 years	31 (5.6%)	15 (16.7%)	7 (3.6%)	9 (3.3%)	
45 < Age ≤ 55 years	67 (12.1%)	15 (16.7%)	19 (9.8%)	33 (12.2%)	
55 < Age ≤ 65 years	174 (31.4%)	27 (30.0%)	61 (31.4%)	86 (31.7%)	
65 < Age ≤ 75 years	161 (29.0%)	21 (23.3%)	58 (29.9%)	82 (30.3%)	
Age ≤ 75	105 (18.9%)	6 (6.7%)	44 (22.7%)	55 (20.3%)	
Age (years)					
Mean ± SD	63.9 ± 12.9	56.1 ± 13.7	65.6 ± 12.4	65.2 ± 12.2	0.000 (Intervention vs. Pre_inter)
Median ± IQR	64.5 ± 15.2	58.4 ± 20.6	65.8 ± 13.9	65.2 ± 14.5	0.000 (Post_inter vs. Pre_inter)
Skewness (Shapiro–Wilk normality)	−0.5 (0.000)	−0.3 (0.272)	−0.6 (0.001)	−0.5 (0.001)	0.712 (Post_Inter vs. intervention)
Range	22.3 ~ 100.5	22.3 ~ 88.2	27 ~ 100.2	24.7 ~ 100.5	(Pairwise proportion test)
Race: *n* (%)	*n* = 550	*n* = 87	*n* = 193	*n* = 270	*0.043 (Pearson's *χ* ^2^)
White/Caucasian	325 (59.1%)	62 (71.3%)	111 (57.5%)	152 (56.3%)	
Black/African American	193 (35.1%)	22 (25.3%)	63 (32.6%)	108 (40.0%)	
Other	32 (5.8%)	3 (3.4%)	19 (9.8%)	10 (3.7%)	
Ethnicity: *n* (%)	*n* = 546	*n* = 83	*n* = 192	*n* = 271	0.347 (Fisher's exact)
Not Hispanic, Latino/a, Spanish origin	520 (93.7%)	80 (88.9%)	178 (91.8%)	262 (96.7%)	
Hispanic, Latino/a, Spanish origin	18 (3.2%)	1 (1.1%)	10 (5.2%)	7 (2.6%)	
Decline to answer	17 (3.1%)	9 (10.0%)	6 (3.1%)	2 (0.7%)	

* Statistically significant differences.

**TABLE 2:** Area deprivation index (ADI) score comparison across phases of intervention

ADI score by study phase	Mean diff (95% CI)	Significance
TelehealthPhase: Intervention vs. preintervention	4.752 (−2.220–11.723)↑	0.181
TelehealthPhase: Postintervention vs. preintervention	7.662 (1.002–14.323)↑	*0.024
TelehealthPhase: Postintervention vs. intervention	2.911 (−2.254–8.075)↑	0.269

* Statistically significant.

**TABLE 3:** Health literacy score in those patients successfully completing telehealth visits in each phase of intervention

	Preintervention	Intervention	Postintervention
Low	6 (6.7%)	33 (17.0%)	30 (11.1%)
Medium	14 (15.6%)	38 (19.6%)	53 (19.6%)
High	53 (58.9%)	103 (53.1%)	145 (53.5%)
No answer	17 (18.9%)	20 (10.3%)	43 (15.9%)
Sum	90 (100.0%)	194 (100.0%)	271 (100.0%)

## 4 DISCUSSION

Creation and deployment of personalized telehealth support, provided via the TTF, among cancer patients significantly increased use of TH for older adults, underrepresented minorities, patients with lower socioeconomic status and those with low HL. The COVID‐19 Pandemic created an exponential increase in TH that will assuredly continue; effective strategies to close the ‘digital divide’ must be researched, deployed and sustained or we will further exacerbate adverse health outcomes and access disparities.^20^, ^21^ Utilization of digital health technology resources like e‐PROs (electronic patient reported outcomes) and online patient portals requires a combination of internet access, sufficient digital and health literacy.^22^ This research calls out the need for future studies to examine digital media literacy and accessibility including; access to smartphone/tablet devices and email, understanding of downloadable applications, and patient willingness and trust in utilizing digital health technologies including TH and e‐PROs for their cancer care.

Our foundational understanding of the impact of targeted TH interventions can help inform future research and clinical deployment of educational interventions, including TH educational materials, and electronic health record orientation. Further efforts will be made to connect patients to digital health options early and often throughout their cancer care, especially as a means to make healthcare more accessible in times of need (i.e. seasonal illnesses, post‐procedure, natural disaster).

Our research shows that cancer patients from vulnerable populations are able to, with the appropriate supports; develop new technological skills and confidence in using TH and multiple digital platforms and applications to communicate with their providers. It should be noted that the SKCC was committed to ensuring access to TH resources for all patients and to that end allocated appropriate human and financial resources during the time of this study without which this would not have been possible. Additionally, because of COVID‐19, many communities have looked at similar disparities among their general populations and access to needed technology resources to support education as well as access to healthcare. Philadelphia recently completed a Household Internet Assessment Survey.^23^ Which gives a broader picture of the challenges faced in our region and provides opportunities for collaboration around systemic interventions to address digital literacy, access and readiness more comprehensively which is ultimately needed. Thomas Jefferson University and The SKCC are collaborators in this report.

## 5 LIMITATIONS

This was a retrospective study, and thus findings are limited as there was no control. In addition, data are only included for patients who successfully used TH. During this time period 3% (36/1120) patients were still unwilling or unable to use TH despite access to the TTF. While this is a relatively small number, we do not know more about this population to understand what additional needs were not addressed. Inclusion of these perspectives in future work is important to ensure digital equity across all popualtions. Also, though translation services are largely accessible and used across the SKCC, the institutional patient portal is currently only available in English and is therefore inaccessible to patients who are not able to understand written English or who did not have access to in‐person assistance due to COVID‐19 quarantine.

## AUTHOR CONTRIBUTIONS

Brooke Worster: conception, design, manuscript preparation; Lauren Waldman: data collection, manuscript preparation; Gregory Garber: conception, design, data collection, manuscript preparation; Tingting Zhan: data analysis; AnaMaria Lopez: conception, design; Olivia Trachtenberg: project administration; Nathan Handley: conception design, manuscript preparation; Kristin Rising: conception, design, manuscript preparation; Valerie Csik: conception, design; Amy Leader: manuscript preparation. All authors reviewed the results and approved the final version of the manuscript.

## CONFLICT OF INTEREST

No disclosures to report.

## Data Availability

The data that support the findings of this study are available from the corresponding author upon reasonable request.

## References

[1] American Cancer Society . Cancer Treatment & Survivorship Facts & figures 2019–2021. American Cancer Society; 2019.

[2] Temel JS , Greer JA , Muzikansky A , et al. Early palliative care for patients with metastatic non‐small‐cell lung cancer. N Engl J Med. 2010;363:733‐742. 20818875 10.1056/NEJMoa1000678

[3] Basch E , Deal AM , Kris MG , et al. Symptom monitoring with patient‐reported outcomes during routine cancer treatment: a randomized controlled trial. J Clin Oncol. 2016;34:557‐565. 26644527 10.1200/JCO.2015.63.0830 PMC4872028

[4] NCI Dictionary of Cancer Terms. Last accessed on November 19, 2019: https://www.cancer.gov/publications/dictionaries/cancer‐terms/def/supportive‐care.

[5] Selby P , Popescu R , Lawler M , et al. The value and future developments of multidisciplinary team cancer care. Am Soc Clin Oncol Educ Book. 2019;39:332‐340. 31099640 10.1200/EDBK_236857

[6] World Health Organization : Palliative Care Fact Sheet, Cancer: Last accessed December 17, 2019: http://www.who.int/cancer/palliative/definition/en/.

[7] Ferrell B , Sun V , Hurria A , et al. Interdisciplinary palliative care for patients with lung cancer. J Pain Symptom Manage. 2015;50:758‐767. 26296261 10.1016/j.jpainsymman.2015.07.005 PMC4666729

[8] Haun MW , Estel S , Rucker G , et al. Early palliative care for adults with advanced cancer. Cochrane Database Syst Rev. 2017;6:CD011129. 28603881 10.1002/14651858.CD011129.pub2 PMC6481832

[9] Ko C , Chaudhry S . The need for a multidisciplinary approach to cancer care. J Surg Res. 2002;105:53‐57. 12069502 10.1006/jsre.2002.6449

[10] Laetz T , Silberman G . Reimbursement policies constrain the practice of oncology. JAMA. 1999;266:2996‐2999. 1820471

[11] Riba MB , Donovan KA , Andersen B , et al. Distress management, version 3.2019, NCCN clinical practice guidelines in oncology. J Natl Compr Canc Netw. 2019;17:1229‐1249. 31590149 10.6004/jnccn.2019.0048 PMC6907687

[12] Donovan KA , Grassi L , McGinty HL , et al. Validation of the distress thermometer worldwide: state of the science. Psychooncology. 2014;23:241‐250. 25160838 10.1002/pon.3430

[13] Hui D , Elsayem A , De La Cruz M , et al. Availability and integration of palliative care at US cancer centers. JAMA. 2010;303:1054‐1061. 20233823 10.1001/jama.2010.258 PMC3426918

[14] Adelson K , Paris J , Horton JR , et al. Standardized criteria for palliative care consultation on a solid tumor oncology service reduces downstream health care use. J Oncol Pract. 2017;13:e431‐e440. 28306372 10.1200/JOP.2016.016808

[15] Butler SF , Fernandez K , Benoit K , et al. Validation of the revised screener and opioid assessment for patients with pain (SOAPP‐R). J Pain. 2008;9:360‐372. 18203666 10.1016/j.jpain.2007.11.014 PMC2359825

[16] Yasin JT , Leader AE , Petok A , et al. Validity of the screener and opioid assessment for patients with pain‐revised (SOAPP‐R) in patients with cancer. J Opioid Manag. 2019;15:272‐274. 31637679 10.5055/jom.2019.0512

[17] Catt S , Starkings R , Shilling V , et al. Patient‐reported outcome measures of the impact of cancer on patients' everyday lives: a systematic review. J Cancer Surviv. 2017;11:211‐232. 27834041 10.1007/s11764-016-0580-1 PMC5357497

[18] Mitchell AJ , Vahabzadeh A , Magruder K . Screening for distress and depression in cancer settings: 10 lessons from 40 years of primary‐care research. Psychooncology. 2011;20:572‐584. 21442689 10.1002/pon.1943

[19] Knies AK , Jutagir DR , Ercolano E , Pasacreta N , Lazenby M , McCorkle R . Barriers and facilitators to implementing the commission on cancer's distress screening program standard. Palliative & Supportive Care. 2019;17(3):253‐261. 29880068 10.1017/S1478951518000378 PMC6286692

[20] Loscalzo M , Clark K , Dillehunt J , Rinehart R , Strowbridge R , Smith D . SupportScreen: a model for improving patient outcomes. J Natl Compr Canc Netw. 2010;8(4):496‐504. Retrieved Oct 21, 2019, from. https://jnccn.org/view/journals/jnccn/8/4/article‐p496.xml

[21] Buxton D , Lazenby M , Daugherty A , et al. Distress screening for oncology patients: practical steps for developing and implementing a comprehensive distress screening program. Oncology Issues. 2014;29(1):48‐52.

[22] American Society of Clinical Oncology . (2021). ASCO Patient‐Centered Cancer Care Certification. Retrieved January 25, 2022, from https://www.asco.org/sites/new‐www.asco.org/files/content‐files/advocacy‐and‐policy/documents/2021‐CertificationPilotOMH.pdf

[23] Centers for Medicare and Medicaid Services . (2021). OCM Quality Measures Guide. Retrieved January 25, 2022, from. https://innovation.cms.gov/innovation‐models/oncology‐care

[24] Cunningham AJ , Edmonds CV . Group psychological therapy for cancer patients: a point of view, and discussion of the hierarchy of options. Int J Psychiatry Med. 1996;26(1):51‐82. 8707456 10.2190/6PG2-GUN8-GK2W-CXAP

